# Supplementary material for: Are carbon emissions trading and green financial instruments synergistic? -Comprehensive quantitative research based on content analysis
Source: PLoS One. 2024 Mar 7;19(3):e0298601. doi: 10.1371/journal.pone.0298601 (PMC10919601; doi:10.1371/journal.pone.0298601)
Supplement: S1 Data — (ZIP) [file pone.0298601.s001.zip › Data/地级市Z_F工作报告.zip!/╣π╢1⁄2/╤⌠╜¡╩╨/└┤╫╘╣1⁄2╓┌║┼í╛┬φ┐╦╩2╛▌═°í┐_2003.docx]

各位代表：

　　我代表市人民政府向大会作政府工作报告，请予审议，并请政协委员和其他列席人员提出意见。

　　　　　　　　　　　　　　　2002年工作回顾

　　2002年是我市经济社会发展取得新成绩的一年。一年来，市政府认真贯彻执行中共中央、国务院的方针政策，在省委、省政府和市委的正确领导下，在市人大及其常委会和市政协的监督支持下，高举邓小平理论伟大旗帜，全面贯彻“三个代表”重要思想，以建设现代化海滨城市为总目标，按照抓好“六个做”、打好“三张牌”的经济工作思路，坚持以发展为第一要务，采取一系列措施，加快发展区域特色经济，全市国民经济保持快速健康发展，改革开放、社会各项事业和精神文明建设取得新成果，顺利完成了市三届人大四次会议确定的各项任务。

　——国民经济保持良好发展势头，经济综合实力上了一个新台阶。2002年，全市国内生产总值首次突破200亿元，共完成205.25亿元，比上年增长11.4%，高出预期目标0.9个百分点，人均国内生产总值7960元，增长10.9%。税收和财政收入大幅增加，地方一般预算收入5.37亿元，按可比口径计算，增长25.7%，全市可支配财力为13.87亿元，收支保持平衡。金融运行平稳，年末全市金融机构各项存款余额168.02亿元，贷款余额97.17亿元，分别比上年底增长11.47%和8.82%。经济结构调整取得新成效，二、三产业增加值分别增长16.2%和11.8%，三次产业增加值比重由上年的36.5：31.8：31.7转变为34.8：33.9：31.3。城市化水平从第四次人口普查的16.98%提高到第五次人口普查的41.92%。

　　——区域特色经济进一步发展。通过加强分类指导，进一步推动区域特色经济的发展。“工业发展活动年”成效明显，特色工业发展加快。全市完成工业总产值191亿元，增长15%。规模以上工业企业产品产销率97.7%，经济效益综合指数为108，比上年提高6.1个点。民营工业已成为拉动工业增长的主要力量，全年完成产值154亿元，增长18.6%，占全市工业总产值的80.6%。成功举办了第一届中国（阳江）国际刀剪博览会，26个国家和地区300多厂家1000多客商同场交流；组建了阳江十八子、广东宏鹰和阳西张氏永光三家民营五金刀剪企业集团；成立了阳江五金刀剪检测中心；五金刀剪行业又一国家级品牌“中国小刀中心”落户我市，“中国指甲钳中心”从上海南移阳江；五金刀剪产值近50亿元，约占民营工业产值的三分之一。食品、服装、家具、建材、机电、化工、医药等行业有新的发展，各类工业园区开发建设势头良好。农业结构进一步调整，农业产业化经营稳步推进。全年农业总产值112.29亿元，增长6.9%。粮经比例由上年的60.3：39.7调整为57.9：42.1，2个国家级、3个省级现代农业示范基地和4个国家级农业综合开发示范项目建设进展顺利。新增水果6.61万亩，水果种植面积共118.16万亩，总产量21.54万吨。完成造林面积16.6万亩。全年肉类总产量10.95万吨，增长6.83%。目前共有省级农业龙头企业3家，省级农业龙头企业培育对象2家，市级农业龙头企业7家。农贸市场建设和管理进一步加强，全市共有农产品流通组织922个，流通人员4.9万人。海洋渔业稳步增长。远洋渔业不断拓展，海水养殖规模进一步扩大，水产品加工和流通有新发展，谊林海达速冻水产有限公司成为目前粤西地区最大的水产品加工企业。国家级中心渔港闸坡渔港和其他6个重点渔港的改造、建设工作抓紧进行。全年水产品总量83.77万吨，增长4.08%；渔业总产值39.81亿元，增长7.2%。旅游业取得新进展。加强重点景区景点建设，一批新的旅游项目建成投入使用，重视抓好旅游宣传促销，举办了第三届阳江旅游文化节。顺利通过“中国优秀旅游城市”复检验收，山海生态休闲旅游品牌逐渐形成。全年接待游客230万人次，旅游总收入11.67亿元，分别增长17.5%和6.45%。

　　——各项改革不断推进。按照中央和省的部署，积极稳妥推进政府机构改革。认真开展行政审批制度改革，对市政府各部门原有的审批事项初步进行了清理和规范，已废止155份不符合WTO规则的规范性文件，取消审批、审核事项141项，政府职能逐步向经济调节、市场监管、社会管理和公共服务等方面转变。启用市投资服务管理中心新办证大厅，进一步优化“一站式”服务环境。经营性土地使用权实行公开招标拍卖，国家投资建设工程、政府采购公开招投标。积极探索建立公共财政体制，行政事业单位工资统发和基本建设项目财政性资金集中支付工作进一步推进。国有企业改革进一步深化，完成7家市直国有工业企业和10家商贸企业的改革工作。个体、私营等非公有制经济和混合型所有制经济快速发展，成为我市经济主要增长点。社会保障制度改革进一步完善，全年企业养老、失业参保人数分别比上年增加2.8%和2.4%。医疗卫生体制和药品生产流通体制改革以及其他各项改革稳步推进。

　　——对外经济贸易取得新进展。积极抓住我国加入世贸组织的新机遇，进一步优化出口商品结构，支持一批民营生产型企业自营进出口，开拓多元化国际市场。市主要领导亲自带团到俄罗斯、日本等国家开展经贸活动。全年完成外贸出口总额4.97亿美元，增长27.4%。其中三资企业和私营企业产品出口分别增长61.8%和236.4%。口岸进出口货运总量55.4万吨，增长89.6%。投资软环境进一步改善，招商引资力度加大，利用外资工作取得新成绩，全年实际吸收外商直接投资首次超过1亿美元，增长27.9%。进一步加强与港澳台的经贸合作，与澳门特别行政区经济财政司开展互访活动。外事、侨务、台务工作为我市的对外开放作出了积极贡献。

　　——国内市场开拓成效明显。继续采取“政府搭台、企业唱戏”的有效办法，组织企业参加南京跨国零售集团国际采购会、全国闲置物资商品交易会、广东—辽宁经济贸易合作洽谈会、第四届中国汕头国际食品博览会等经贸活动，积极拓展国内市场。市内市场繁荣兴旺，居民消费结构逐步升级，房地产、汽车、信息等消费市场升温。全年商品房完成销售面积18.72万平方米，销售额2.44亿元。餐饮业增长12.9%。通过整治涉农收费，改造农村电网，提前半年实现农村到户电价0.79元/千瓦时，切实减轻农民负担，改善农村消费环境。全市消费品零售总额96.48亿元，增长12%，剔除价格因素，实际增长15 %，消费需求拉动国内生产总值增长7.1个百分点，成为我市经济增长重要的拉动力量。

　　——基础设施和城市建设迈出新步伐。全年完成全社会固定资产投资46.61亿元，增长10.2%，增幅比上年提高9.6个百分点。开阳高速、阳茂高速、沿海高速、省道113线阳春南段、阳阳铁路等15个重点项目完成投资12.38亿元，超过年初预期目标。房地产开发完成投资8.95亿元，增长36.43%。新增国债资金9660万元。非国有投资完成29.5亿元，增长24.2%，占全社会固定资产投资的63.3%。市委、市政府提出的为民办实事项目进展顺利，市汽车总站、中国刀具城会展中心等项目相继建成投入使用；马南垌排洪整治工程、鸳鸯湖景区、创业路中段、南恩路一期改造工程、城区和农村电网年度改造计划完成或基本完成；漠江中路、安宁路、文化广场、世纪公园、阳江职业技术学院教学大楼、市区燃气管道工程等项目已完成前期工作进入施工建设。城市环境整治力度加大，市容市貌进一步改观。水利大会战一期工程顺利完成，二期工程顺利推进，修建、加固江海堤围15.6公里，完成小型水库除险加固工程87宗。投入资金近3000万元，建成通行政村水泥路23条，115公里。

　　——科教事业加快发展。实施科技创新9项计划，进一步抓好高新技术和先进适用技术的推广应用，改造提升传统产业。以企业为主体的技术创新体系逐步建立，在组建10家市级工程技术开发中心的基础上，经省批准成立了3家省级工程技术研究开发中心，新增4家省级高新技术企业和14家省级民营科技企业，全市共有省级高新技术企业11家，省级民营科技企业56家。全年专利申请量425项，比上年增长50 %。高新技术产品产值26.9亿元，占工业总产值的14.2%。科技进步对工业经济增长的贡献率为45.6%。经省政府批准在市直工业开发区的基础上兴办阳江高新技术产业开发区，作为我市发展高新技术产业的中心基地、科技成果转化和改造传统产业的示范基地、招商引资和做大工业的重要载体。“普九”教育进一步巩固提高，高中阶段教育加快发展，成人教育、职业教育和高等教育稳步发展。“以县为主”的基础教育管理体制基本确立。中小学“一费制” 全面实施，对32467名农村困难家庭子女免收义务教育阶段书杂费。合理调整中小学布局，撤并112所中小学校分教点，改扩建121所中小学，易地新建15所中小学，改造老区小学62所，有3所学校晋升为省一级学校。人才队伍建设进一步加强，构筑人才交流平台，全年举办14次人才交流活动，为企业和单位引进、输送了一批专业人才。

　　——民主法制建设得到加强。坚持和完善向人大及其常委会报告工作、向政协和各民主党派通报情况的制度，自觉接受监督。认真办理人大代表议案、建议和政协提案。城乡基层组织建设和民主政治建设不断加强，村民委员会换届选举进展顺利，村务公开、厂务公开扎实推进，政务公开取得较好成效，省在我市召开政务公开现场会，对我市政务公开工作给予充分肯定。推进依法行政，规范行政执法主体及其行为，行政执法责任制和执法过错责任追究制进一步落实。认真抓好领导干部廉洁自律、查处大案要案、纠正部门和行业不正之风以及从源头上预防和治理腐败等工作，廉政建设取得良好成效。普法教育、法律服务工作得到加强。审计、监察、统计等工作更好地为经济社会发展服务。整顿和规范市场经济秩序工作取得阶段性成果，打击偷税骗税、制售假冒伪劣商品、私宰生猪和整治加油站、集贸市场工作取得新成绩，重视市场物价监管，维护消费者利益，进一步加强信用建设，市场环境有效改善。高度重视安全生产，安全生产形势保持稳定。切实抓好反走私斗争。深入开展严打整治斗争，加强社会治安综合治理，严厉打击各种严重刑事犯罪活动，扫除“黄赌毒”，坚决取缔“法轮功”邪教组织。全市社会治安稳定，刑事案件下降24.6%。

　　——精神文明建设和社会各项事业全面进步。进一步健全社会主义精神文明建设的领导体制和工作机制。深入开展学习邓小平理论、贯彻“三个代表”重要思想和党的十六大精神活动。全面实施《公民道德建设实施纲要》。以创建省文明城市为龙头，广泛开展群众性精神文明创建活动，公民素质和城市文明程度进一步提高。新闻出版、广播电视事业不断发展，群众文化娱乐活动丰富多彩，文艺创作日趋活跃，文化市场健康发展，有两个戏剧获全省第四届群众戏剧花会银质奖，11项山区文化建设工程顺利完成。城乡爱国卫生、医疗服务、疾病控制和药品监督工作进一步加强。创建省卫生城市活动顺利通过考核验收，我市被省爱卫会授予“广东省卫生城市”和“广东省灭蝇先进城区”称号。积极开展中国儿童慈善一日捐活动，我市被授予“中国公益明星城市”。全民健身运动深入开展，竞技体育水平有所提高，共获得省级以上体育奖牌97枚，其中在省第十一届运动会上获得了4.5枚金牌、10枚银牌和9枚铜牌，取得了历史最好成绩，实现了预期目标。人口与计划生育工作扎实开展，人口自然增长率下降到6.55‰，再次获得省政府的通报表彰。殡葬改革力度加大，受到省政府的表彰。国土资源保护工作进一步加强，土地用途管制制度全面实施，土地后备资源开发力度加大，耕地占补实现平衡。创建“全国双拥模范城”工作扎实推进，军政军民团结进一步加强，全民国防教育广泛开展，人民武装和民兵预备役工作取得新成绩。民政、民族、宗教、妇女儿童、人防、气象、地震、档案、文史等各项工作都有新发展。

　　——城乡人民生活水平进一步提高。2002年农村居民人均纯收入3940元，比上年增长3.7%；城镇在岗职工人均工资收入9165元，增长4.97%。城乡居民最低生活保障制度逐步健全，低保保障面进一步扩大，享受低保对象由原来的1.8万人增加到3.33万人，基本实现应保尽保。提高了企业离退休人员养老金标准，确保国有企业下岗职工和企业离退休人员养老金按时足额发放。采取得力措施帮助困难群众解决住房难、就医难、打官司难、子女入学难等问题。积极推进农村贫困户泥砖房改造工作，有518户困难群众建新房1113间。认真抓好就业和再就业工作，城镇登记失业率为3.2%，就业形势保持稳定。环境资源保护和生态建设得到加强，生活环境进一步改善。

　　各位代表，过去一年我市国民经济和社会发展取得的成就，是省委、省政府和市委正确领导的结果，是各级人大及其常委会和政协监督支持的结果，是全市党政军民努力实践邓小平理论和“三个代表”重要思想，坚持把发展作为第一要务，迎难而上，艰苦奋斗，与时俱进，开拓创新的结果。在此，我代表市人民政府，向全市广大工人、农民、知识分子、干部职工、驻阳江人民解放军、武警部队官兵、人民警察和各界人士致以崇高的敬意！向长期关心支持我市建设的香港、澳门特别行政区同胞、台湾同胞、海外侨胞和国际友人表示衷心的感谢！

　　我们清醒地看到，成绩来之不易，差距仍然很大。目前我市经济社会发展中存在的突出问题是：产业结构仍不合理，工业的扩张和升级力度不够，产业竞争力不强；在建的重大建设项目不多，后备项目储量不足；农民和干部职工收入增长缓慢，部分群众生活比较困难；高素质人才资源缺乏，劳动力供求结构性矛盾较突出，劳动就业压力加大；资金困难，财政收支平衡能力弱；投资软硬环境有待进一步优化，招商引资工作未能取得突破性进展；整顿规范市场经济秩序和社会治安秩序任务繁重；精神文明建设和民主法制建设还存在不少薄弱环节等。对这些问题和困难，我们必须高度重视，进一步采取有力措施，认真加以解决。

　　　　　　　　　　　　　　　　2003年工作安排

　　今年是贯彻落实党的十六大精神、全面建设小康社会的重要一年。我市经济社会发展所面临的环境既有许多有利条件，也有不少新情况新问题。总的来说是机遇大于挑战，但由于我市仍是相对落后地区，面临的困难仍然比较突出。我们必须认清形势，解放思想，把握机遇，与时俱进，始终坚持把发展作为第一要务，始终保持良好的精神状态和务实的工作作风，迎难而上，开拓创新，在创新中提高，在提高中发展，加快发展区域特色经济，努力增强经济实力和发展后劲，为全面建设小康社会打下良好的基础。

　　今年市政府工作的指导思想是：以邓小平理论和“三个代表”重要思想为指导，全面贯彻落实党的十六大和省第九次党代会精神，按照省委九届二次全会和市委三届九次全会的部署，以全面建设小康社会和建设现代化海滨城市为总目标统揽工作全局，以发展为主题，以结构调整为主线，以改革开放和科技进步为动力，以提高人民生活水平为根本目的，继续实施“追赶型、科教兴市、可持续发展”三大战略，坚持抓好“六个做”，打好“三张牌”，增创区域特色经济新优势，促进国民经济持续快速健康发展，正确处理改革发展稳定的关系，大力加强社会主义政治文明和精神文明建设，推进社会各项事业全面进步，努力开创各项工作新局面。

今年全市经济发展主要预期目标是：国内生产总值增长11.5%，农业总产值增长5.8%，工业总产值增长15%，外贸出口增长8%，实际利用外资增长10%，固定资产投资增长12%，社会消费品零售总额增长12 %，地方财政一般预算收入增长10%，城镇登记失业率控制在3.5%以下，人口自然增长率控制在8.3‰以内，城镇在岗职工人均工资增长5.5%，农村居民人均纯收入增长3.5%。

　　为实现上述目标，必须做好以下工作：

　　一、加快经济结构调整步伐，进一步做大工业、做优农业、做强海洋产业、做旺旅游业

　　走新型工业化道路，大力做大工业。坚持“工业兴市”方针，继续深入开展“工业发展活动年”活动，以加快工业化为核心和主攻方向，真正把发展工业作为经济工作的重中之重，努力把特色工业做大做强，重点企业做优做新，名牌产品做精做专，推动传统产业在实现量的扩张的同时促进质的提高，积极发展对经济增长有突破性带动作用的高新技术产业。努力办好阳江高新技术产业开发区和各类工业园区。按照十六大提出的以信息化带动工业化，以工业化促进信息化的要求，以高新区为依托，争取在发展电子信息、生物工程、新材料和环保产业等方面取得突破，促进工业结构优化升级。加快用高新技术和先进适用技术改造提升传统工业。切实抓好五金刀剪、食品加工、建材机电、纺织服装、医药化工、家具编织等重点行业的技术改造，鼓励和支持企业加快技术创新步伐，提高自主创新能力，增加自主知识产权拥有量，增强核心竞争力。重视发展中小企业和农产品、水产品等资源加工型工业。建立中小企业管理机构，健全面向中小企业的社会服务体系。鼓励内资、外资、民资投到办工业上，上马一批新工业项目；鼓励“十八子”、“银鹰”、“永光”、“宝马利”等企业加快技术改造步伐，提升企业素质和竞争力；鼓励“喜之郎”、“丰源粮油”、“谊林海达”等食品加工企业增资扩产，做强品牌；支持阳春家具制造业的发展，做大特色产业。实施名牌带动战略，继续推行国际标准认证，改进产品包装，鼓励企业瞄准国际国内市场，创名牌、出精品，对获得中国驰名商标、中国著名商标和广东省名牌产品的企业给予物质奖励。积极配合阳江核电站建设前期工作，发挥重大项目的综合效应，拉动我市经济发展。

　　加快农业结构调整，推进产业化经营，努力做优农业。认真贯彻“多予、少取、放活”的方针，千方百计增加农民收入。稳定和完善农村土地承包政策，规范农户承包土地经营权有序流转。转变农业增长方式，引导农民以市场为导向，以效益为中心，进一步调整种养结构，发展优质、高产、高效、生态、安全农业。扩大优质稻和亚热带优质水果、蔬菜、蚕桑、南药、花卉等种植面积，大力发展无公害农产品、绿色食品，继续扶持和发展瘦肉型猪、山地鸡、黄鬃鹅、沿海蛋鸭、肉牛和奶牛等畜禽生产。办好市农产品质量检验测试中心，推进农业标准化和农产品质量安全体系建设，打造具有地方特色的双肩玉荷包荔枝、上洋西瓜、阳春马水桔、春砂仁等“名、优、特”品牌，发展效益农业和创汇农业。加快农业先进适用技术推广应用，健全农业技术推广、信息发布和技术培训网络。鼓励有条件的地方发展立体农业、生态农业、休闲农业。搞活农产品流通，大力抓好农产品市场和流通组织建设。积极培育农业龙头企业，推进农业产业化经营。重点扶持14家市级以上农业龙头企业，办好10个农产品专业市场，增强农业龙头企业带动农户的能力。大力发展“订单农业”、合作经济组织和各类行业协会，谋求经营机制创新，提高农业生产组织化程度。推进农业现代化建设步伐，认真抓好2个国家级、3个省级和3个市级农业现代化示范区建设。加大农业综合开发力度。促进乡镇企业体制创新，增强农村集体经济实力。全面推进涉农价格、收费公示制和中小学“一费制”，按省的部署稳妥开展农村税费改革，减轻农民负担。加强农业基础设施建设，继续抓好行政村通水泥路和农村电网改革改造工作，全面完成全市水利大会战确定的75宗小型水库和6宗堤围加固达标工程，继续实施漠阳江排洪防涝工程，加强农村机电排灌工程建设，提高防汛抗旱能力。加大农村环境卫生治理力度，减少生产、生活垃圾造成的污染，提高农村环境质量。

　　加大产业升级力度，切实做强海洋产业。坚持以市场为导向，以科技为依托，主攻养殖业，拓展远洋捕捞业，深化加工业，搞活流通业，努力在提高水产品附加值和海洋渔业经济效益上取得新的突破。充分利用浅海滩涂、低产田、咸酸田发展养殖业，重点扶持发展一批科技含量较高、规模较大的海水养殖基地，进一步提高养殖业在海洋渔业中的比重。继续抓好远洋捕捞基地建设。突出抓好水产品深加工和流通，扶持发展一批渔业龙头企业，着力培育一批水产品知名品牌，提高国内外市场占有率。加快市水产品中心市场的建设，努力把它建成一个集水产品交易、渔需物资供应和渔业信息处理于一体的现代化、智能化大型综合市场，提高渔业产业化水平。抓好闸坡国家中心渔港和其他重点渔港的改造建设，进一步完善配套服务功能。认真解决渔业、渔民转产后出现的实际问题。大力发展人工渔礁，建立休闲渔业基地。积极发展临海工业、滨海旅游业、观光渔业、海洋药物、海洋保健食品和海上运输业，促进海洋资源的综合开发。

　　整合资源，突出特色，进一步做旺旅游业。坚持规划先行，重点带动，打造品牌，突出特色。抓好全市旅游总体规划的修编工作，做好与城市发展总体规划和景区详细规划的衔接，确保旅游资源依法有序开发。突出山海生态休闲旅游特色，以海陵岛为龙头，整合山海湖泉林洞旅游资源，形成五大旅游组团（江城至海陵岛、春湾凌霄岩、八甲、阳东、阳西），提高“阳江游”知名度。促进旅游开发与城镇建设一体化，加快重点旅游景区景点、人文景观和基础设施建设。争取启动“广东海上丝绸之路博物馆”建设。下功夫开发一批具有阳江特色和文化内涵的旅游商品、纪念品，建设大型旅游购物商场。加大招商引资力度，吸引外商投资高尔夫球场和高等级宾馆，进一步完善食、住、行、游、购、娱配套建设。建立市旅游指导服务中心，为游客旅游、住宿、餐饮、购物等提供咨询服务，营造良好的旅游环境。举办第四届阳江旅游文化节，更好地促进旅游、文化、体育、会展活动的融合，推动休闲产业、文化产业的发展，提高综合效益。

　　二、大力推动民营经济发展提高，进一步深化国有企业改革

　　促进民营经济在创新中发展，在发展中提高。民营经济是我市经济的重要支柱，要把发展民营经济作为扩大经济规模、增强经济实力和发展后劲的关键点来抓，对民营企业实行“政治平等、政策公平、法律保障、放手发展”的方针，努力为民营企业创业人员营造“社会上有地位、政治上有荣誉、经济上有实利”的良好发展氛围。进一步完善中小企业和民营企业管理机构，加强对民营企业的引导和服务。放宽市场准入条件，凡符合国家法律法规、产业政策规定以及加入世贸组织后对内对外开放的行业和投资领域，都对民营企业开放。对所有企业申请注册登记、自营进出口权、进出口配额等都一视同仁。加大依法保护力度，维护民营企业合法权益。改善金融服务，逐步解决民营企业融资难问题。进一步健全民营企业社会服务体系，为民营企业提供信息、技术、人才、法律等多方面服务。设置民营企业展示中心，为科技成果产业化、成熟产品找市场和招商引资提供“互动平台”。积极发展民营科技园，支持科技型、外向型、农业型、扶贫型和劳动密集型民营企业发展，力争今年我市省级民营科技企业突破100家。大力引导民营企业进行观念创新、制度创新、人才创新、技术创新和产品创新。在推进五金刀剪行业集团化、规模化生产的同时，在食品加工、纺织服装、家具、化工、医药等行业培育扶持一批民营企业集团。重视民营企业技术改造，加强产品质量检验检测工作，创建五金刀剪“阳江标准”，支持“十八子”、“银鹰”、“永光”争创中国驰名商标，促进民营企业在注意量的扩张的同时更加重视质量和效益的提高。

　　完善国有资产管理体制，推进国有资产优化重组。进一步健全权利、义务和责任相统一，管资产和管人、管事相结合的国有资产管理体制。完善国有资产保值增值考核办法，推进国有企业分配制度改革。加快放开搞活国有中小企业的步伐，今年内基本完成市属国有工业企业的改制重组，明年扫尾。积极支持民营企业参与国有经济的调整，大力发展股份制企业和混合型经济。

　　三、积极开拓国际市场，努力扩大对外开放

　　实施多元化战略，千方百计增加出口。加快调整和优化出口产品结构，实行科技兴贸。加强技术创新和产品开发，提高出口产品的科技含量和附加值。抓好传统出口商品的升级换代，力争培育一批出口名牌、著名商标。扶持高新技术产品和农渔等特色产品出口。在巩固提高美、日、欧和港澳等传统出口市场的同时，着力开拓俄罗斯、东欧、拉美、非洲等新市场。举办第二届中国（阳江）国际刀剪博览会，推进五金刀剪行业更好地走向世界。大力推动一般贸易，重视发展加工贸易。加快国有外贸企业改革，支持民营企业开展外经贸业务，培育出口龙头企业。加强涉外部门的协调配合，改善外经贸环境，提高通关效率。继续落实出口扶持奖励措施，对生产企业出口货物实行“免、抵、退”政策。协调银企、税企关系，扩大出口信贷，缓解外贸企业资金供求矛盾。

　　加大招商引资力度，提高利用外资水平。进一步优化投资软环境，真正做到“低门槛、宽政策、少收费、善管理、优服务、重信用”，努力降低城市运作成本，减少营商成本，形成良好的综合发展环境，增强对外商的吸引力，力争成为外资和项目流向、聚集的洼地。进一步密切与港澳台的经贸联系与合作，大力拓展与国内尤其是珠三角地区的合作领域，更加积极主动地承接发达地区的产业转移。办好各类工业园区，使之成为招商引资的主要载体。鼓励现有外商企业增资扩产，创造条件吸收美、日、欧等发达国家的资金，争取引进跨国公司、国际大财团到我市投资和设立加工基地、采购中心。推进与粤西地区和周边城市包括珠海、澳门等地的优势互补，促进在工业、农业、服务、物流和旅游等领域的交流与合作。继续落实招商引资责任制，兑现奖励政策，创新招商引资方式，选择重点项目主动出击招商，并把上门招商与以商引商、委托招商、节会招商、网上招商等结合起来，多渠道、全方位开展招商引资。引导外资重点投向高新技术产业、制造业、农（水）产品深加工、现代服务业、基础设施建设和国有企业改造等领域。加强已签约项目的跟踪落实，促使其尽快履约实施，争取今年实际利用外资有较大的突破。

　　四、发展现代流通业，大力拓展国内市场

　　加快发展现代服务业，促进新消费热点的形成。把加快现代服务业的发展作为提高经济运行质量和效率，优化经济结构，培育新的经济增长点的一个重要途径来抓，积极应用信息技术改造服务业，继续发展商贸流通、交通运输、餐饮、零售等传统服务业，认真抓好信息、咨询、会计、审计、法律、建设监理、房地产等中介服务的规范发展，加快发展市政服务、城镇社区服务和文化、体育产业，促进形成旅游、住宅、汽车、信息服务、智力开发、教育、体育健身、卫生保健等新的消费热点。加快与电子商务配套的认证体系、网上支付体系建设，促进电子商务发展。大力发展连锁经营，鼓励中小商贸流通企业进一步拓展经营范围，并向多业种、多业态发展。成立市展览公司，促进会展业的发展。规范垄断行业和服务业的价格行为，全面推行明码标价和收费公示制。进一步简化消费信贷手续，引导居民消费预期，扩大居民即期消费。

　　加大市场开拓力度，拓展新的市场空间。进一步推进城乡用电同网同价，改善农村消费条件，促进农民消费，扩大农村市场。继续组织企业参加国内的大型展销会、洽谈会、订货会，更好地发挥“政府搭台、企业唱戏”的作用。在抓好传统市场的同时，重视拓展我国西北、西南和东北等新的市场空间。引导企业改变营销策略，积极到全国各地建立销售服务点，同时实行市场开拓和经贸合作相结合，加强与内地企业在资源、品牌和资产等方面的投资合作，提高合作层次，扩大销售网络。

　　五、加大投资力度，推进城市化进程

　　多渠道筹集资金，保持投资稳定增长。继续调整优化投资结构，扩大对有社会需求的公益性、社会性、基础性领域的投资，重点推进一批有利于促进城乡经济发展的交通、能源、通信、水利、城市基础设施等重点项目的建设。抓好阳江核电项目的前期工程，全面完成开阳高速公路阳江段建设，加快阳茂高速公路阳江段、省道113线阳春南段的建设步伐，抓好阳阳铁路收尾工程，加快阳江港3.5万吨多用途码头和3万吨粮食专用码头建设，抓好电信扩建扩容、联通网络改造和网通网络建设工程等。

　　大力加强城市建设和管理，进一步完善城市功能。大力抓好城市发展总体规划的完善，重点抓好市区近期建设规划、重要地段控制性规划和专项规划、小区详细规划的修编，强化规划的严肃性，促进城市建设规范化、法制化。按照完善城市功能、提高城市品位、提升城市形象的要求，突出山水生态休闲旅游城市特色，精心抓好城市景观、主要街景和重点建筑物的设计与建设。抓好石湾北路、高凉路、创业路北段、江台路以及体育路北段、鸳鸯湖外环湖路、河堤南路等道路的建设和改造工作，加快人民广场、文化广场、文化艺术中心、世纪公园的建设步伐，抓好金山森林公园首期工程、汽车物流中心、第一市场改造等项目的建设，推进“一江两岸”整治工程的规划设计和分步建设，抓好景湖花园、愉景花园、湖湾华庭、雨田广场等住宅小区的建设。重视城市解困房和无障碍设施建设。进一步抓好城市的绿化、净化、美化、亮化工作，营造城市亮点。重视市区停车场的规划与建设。制定人民防空工程建设规划，提高城市总体防护能力。深化投资体制改革，对限额以下的一般竞争性项目，凡属于国家鼓励和允许的，原则上将审批制改为登记备案制。树立经营城市理念，加快城市市政公用事业市场化进程，大力吸引外资、内资参与城市开发与建设，促进民间投资的增长，鼓励和引导民资投向交通、能源、通信、供水、环境保护等基础设施、旅游中介服务项目和公用事业建设。加大城市管理力度，改革和完善城市管理体制，实行“两级政府、三级管理、四级网络”，做到条块结合、以块为主、重心下移，加强综合执法，强化 “六乱”整治，美化市区东西出入口，整治交通秩序，落实环卫、绿化等工作责任制，进一步提高城市管理水平。

　　积极探索创新，加快城镇化进程。把推进城镇化建设作为促进城乡、区域协调发展的重大战略举措来抓，以发展县城和中心镇为重点，逐步建设一批基础设施配套完善、各具特色的现代化中心镇，促进二、三产业向城镇聚集，引导农村人口向城镇转移，加速农村城镇化进程。完善城镇规划体系，加大镇、村规划编制力度，统一规划住宅区、工业区、农田保护区和生态区，严格按规划建设。积极探索、改革创新小城镇建设模式，打破体制和政策障碍，用改革的办法解决用地、资金、入户等问题，努力走出一条适应发展要求和符合我市实际的市区、县城、中心镇、建制镇协调发展的城市化路子。

　　六、重视发展教育事业，大力推进科技创新

　　推进教育改革创新，加快教育事业发展。坚持教育优先发展，增加教育投入，深化教育改革，实施素质教育，提高教育质量，培养大批高素质劳动者和实用型、创新型人才。进一步落实农村基础教育“以县为主”的管理体制，鼓励、支持和规范多种形式的社会办学。推进中小学布局调整，扩大城区办学规模，新增改造60所老区、山区小学。重视幼儿教育，巩固提高九年义务教育，加快发展高中阶段教育，推进国家、省级示范高中学校建设，积极发展成人教育和职业教育，整合普通中专、成人中专、职业高中、技工学校等中等职业教育资源，办好高等教育。完善中小学“一费制”和免收农村困难家庭子女义务教育阶段书杂费制度。推进学校定编、定员、定岗和教师聘任制工作，加强教师队伍建设。加快教学手段现代化步伐，推进中小学信息化教育和英语教育，在已完成70所中学配备电脑室的基础上，今年力争为100多所中心小学、县城小学配备电脑室。

　　加快建立技术创新体系，增强科技创新能力。全面实施科技创新9项计划，加快建立以企业为主体的技术创新体系，引导企业特别是骨干企业建立技术开发机构，完善提高10家市级工程技术开发中心，争取新增一批企业建立省级工程技术研究开发中心，做大做强一批具有自主知识产权、核心能力强的科技企业。建立重点面向中小企业的科技服务体系，发挥科技中介服务机构作用，发展技术市场。加快高新区和民营科技园的建设，力争在高新技术产业发展上取得新的突破，提高科技进步对经济增长的贡献率。引导企业增强知识产权保护意识，进一步做好专利申请工作。加强网络资源整合，切实抓好企业信息化应用公共平台和电子商务建设，加快信息技术在各行各业的广泛应用。办好海陵岛“广东省依靠科技进步推动旅游产业发展示范区”。深化科技体制改革，高度重视科技人才队伍建设和人才资源整体开发，认真培养和吸引更多的高层次人才，充分调动广大科技人员的积极性、主动性和创造性。

　　七、做好就业和社会保障工作，改善人民生活

　　做好就业和再就业工作。认真落实下岗职工再就业的各项优惠政策，鼓励企业吸纳下岗失业人员，支持下岗失业人员自谋职业、自主创业。立足于发展经济扩大就业，通过大力发展民营经济、劳动密集型企业以及旅游、商贸、饮食和社区服务等第三产业，创造和扩大就业岗位。引导下岗失业人员转变择业观念，鼓励他们通过非全日制、非固定单位、临时性和弹性工作等灵活多样的形式实现就业。建立就业援助制度，重点扶持大龄再就业困难群体实现再就业。抓好市劳动力中心市场和技工学校建设，加快建立以公益性职业介绍机构为主体的就业服务体系，为下岗失业人员提供职业介绍、就业指导、职业培训等“一站式”服务。利用现有教育资源开展多形式的再就业培训，增强下岗失业人员再就业技能。加强与珠三角等经济发达地区的劳务协作，扩大农村剩余劳动力的就业门路。依法加强劳动用工管理，保障劳动者的合法权益。

　　建立健全与经济发展水平相适应的社会保障体系。进一步完善城镇职工基本养老保险、基本医疗保险、失业保险和城乡居民最低生活保障制度。确保国有企业下岗职工基本生活费和离退休人员养老金按时足额发放。继续抓好下岗职工基本生活保障向失业保险并轨工作。推进企业退休人员社会化管理。将符合条件的城乡贫困人口和长期亏损、停产、半停产的在岗职工纳入最低保障范围，做到应保尽保。加大社会保险费征缴力度，大力清收企业欠缴的社会保险费。进一步健全社会保险基金监管体系，规范保险基金的管理。

　　关心困难群众生活。继续解决好困难群众的“四难”问题。建立健全农村合作医疗保障体系，逐步解决农村人口“就医难”和“因病返贫”等问题。认真解决部分农村群众饮水难问题。继续推进扶贫开发，巩固扶贫成果。帮助城镇现有住房特困户解决住房困难，按计划扎实有效推进农村贫困户泥砖房的改造，关心支持残疾人事业，推进残疾人康复和就业。

　　八、坚持依法治税，强化财政监督

　　加强税收征管，抓好开源节流。加大依法治税力度，深化税收征管改革，继续整顿和规范税收秩序，加大税务稽查力度，依法严厉打击和查处偷、逃、骗、抗税行为。清缴企业欠税。推进私营企业、个体工商户建帐制度。加快建立以信息化、管理专业化为主的现代税收征管模式和机制，进一步提高税收管理水平。

　　健全财政管理制度，强化预算监督。推进建立公共财政体制，确保重点支出，压缩一般性支出，严格预算约束，不准打赤字预算。逐步推进部门预算改革和部门综合预算管理。继续推进国库集中支付制度改革。严格控制使用财政性资金的投资项目，加强国债项目资金管理的检查监督，把好资金使用关。全面实行政府采购制度。厉行节约，严禁公款旅游，大力精减各种会议，切实控制会议费、招待费、差旅费等一般性支出。各级行政事业单位不得自行增设机构，不得超编增人。结合机构改革与农村税费改革，压缩财政供养人员，减轻财政支出压力。

　　整顿财经秩序，严肃财经纪律。严格执行“收支两条线”管理规定。强化审计监督。加大对会计师事务所等中介机构的监督力度。继续推进向国有企业、行政事业单位和重点基建项目委派财务总监工作。坚持依法理财，严厉查处各种违反财经纪律行为。进一步抓好农金会和城信社的资产清收工作。

　　九、加强环境资源保护和计划生育工作，促进可持续发展

　　重视环境保护和生态建设。继续实施漠阳江水质保护规划，建设漠阳江流域生态林工程，抓好漠阳江上游阳春段水污染防治和市区“一江两岸”整治工作，划定镇级集中饮用水源保护区，开展保护区内污染源的清理整治，密切监控饮用水源断面水质状况，确保饮用水源水质安全。推行清洁生产和循环经济，切实治理污染严重行业，推进工业污染源治理全面达标。加大城镇环境综合整治力度，认真治村、治水、治小区，提高城镇污水、生活垃圾和废物集中处理率，加强对生产生活噪声的监管，控制市区摩托车入户数量和行驶范围，改善城镇环境质量。抓好生态示范村（镇、场、园）建设。巩固发展造林绿化成果，提高森林覆盖率。重视自然保护区、森林公园建设和管理，推进鹅凰嶂自然保护区申报省级和国家级自然保护区工作。依法保护和合理开发各种自然资源。严格控制非农用地，实现耕地占补平衡。保护海洋环境和海岸等生态系统。健全环境监测体系，加强地震和气象预测预报，提高抵御自然灾害的能力。

　　提高人口与计划生育工作水平。认真执行《中华人民共和国人口与计划生育法》和新修订的《广东省人口与计划生育条例》，控制人口增长，稳定低生育水平，提高人口素质。落实人口与计划生育目标管理责任制，建立健全计划生育“依法管理、村（居）民自治、优质服务、政策推动、综合治理”新机制。加强对流动人口计划生育的管理与服务，建立以现居住地管理为主的工作机制。重视解决老龄人口社会保障和精神文化生活问题。

　　十、推进文化建设和民主法制建设，发展社会各项事业。

　　以“三个代表”重要思想统领社会主义文化建设。深入宣传贯彻十六大精神。以马克思列宁主义、毛泽东思想、邓小平理论和“三个代表”重要思想为指导，推进文化建设和文化体制改革，积极发展文化事业。探索建立文化产品生产经营的新机制，推进文化与经济融合，发展文化经济和文化产业。改革国有文化企事业单位，整合资源，走集团化发展路子。繁荣文艺创作，促进文化精品的创作生产。积极挖掘和整理历史文化遗产和优秀民间艺术，打造漠阳文化品牌。加强基层文化建设，推进社区文化、企业文化、校园文化等群众文化活动的开展，以乡镇带村庄，实现先进文化“进村入户”，丰富和活跃城乡群众文化生活。坚持依法治市与以德治市相结合，认真贯彻《公民道德建设实施纲要》，积极开展“五四三二一”系列公民道德建设活动，以为人民服务为核心，以爱国主义、集体主义为原则，以诚实守信为重点，加强社会公德、职业道德和家庭美德教育，寓教育于活动，在参与中提高，促进公民道德素质的提高和良好社会风尚的形成。以创建省文明城市为龙头，深入开展多种形式的精神文明创建活动，提高城乡文明程度，抵制封建迷信及其他各种落后、腐朽思想，倡导文明健康的生活方式。

　　推进民主法制建设，建设社会主义政治文明。坚持政府向人大及其常委会报告工作，向人民政协和民主党派通报情况的制度，自觉接受监督。认真办好人大代表议案、建议和政协提案。建立和完善重大决策的规则和程序。发挥民主党派、工商联、无党派人士参政议政作用和工会、共青团、妇联组织的桥梁纽带作用。加强信访工作，进一步落实领导干部接访日制度，继续办好“市长专线电话”，多方听取人民群众的意见和要求，扩大民主决策。健全基层自治组织，完善村民自治和居民自治，搞好村务公开和厂务公开。加强政府法制建设，提高依法行政水平。认真抓好“四五”普法教育，增强公职人员的依法办事能力和公民的法律素质。做好行政复议应诉工作，健全行政执法责任制，强化行政执法监督。进一步清理、修订、废止与世贸组织规则和我国对外承诺不一致的地方政府文件。

　　促进社会各项事业发展。逐步建立与国民经济和社会发展相适应的医疗卫生服务体系和医疗保健体系。重视农村卫生工作，加大对农村卫生的投入，着力改善农村医疗卫生状况，逐步建立农村卫生服务新体系。深化城镇医疗卫生体制改革，完善医疗机构分类管理制度，发展民营医疗机构，试点推行民营医院。加快发展社区卫生服务。建立健全卫生监督执法和疾病预防控制体系，建设市疾病预防控制中心，扶持发展中医药事业。加强医风医德建设，狠抓医疗质量管理。整顿规范药品市场与医疗机构，规范和扩大药品、医疗器械集中招标采购。加强学校食品卫生和工厂企业卫生管理。巩固提高创建广东省卫生城市成果，扎实推进爱国卫生工作。巩固提高无偿献血工作。发展群众体育事业。构建亲民、便民、利民的体育服务体系，推动全民健身活动广泛开展。培养优秀体育人才，提高竞技体育实力。发展体育产业，培育体育市场。提高统计工作服务水平。抓好全民国防教育，增强公民国防意识，提高人民武装和民兵预备役工作水平。以创建“全国双拥模范城”为动力，促进双拥共建和优抚安置工作上新台阶。重视人防工作，完善防空设施建设管理。加强民间组织管理。继续重视和做好外事、侨务、台务、档案、文史等工作。深化华侨农场体制改革，关心归侨生产生活。

　　认真做好民族宗教工作。全面贯彻落实民族宗教政策，尊重和保护公民宗教信仰自由，积极引导宗教与社会主义社会相适应。进一步做好民族工作，推进民族团结进步事业。

　　加快妇女儿童事业发展。认真实施妇女儿童发展规划，切实维护妇女儿童合法权益，保障妇女参政议政、劳动就业等方面的平等权利。提高妇女儿童保健水平，重视儿童培养。

　　十一、加强信用建设，优化发展环境

　　健全社会信用体系。建立完善行政审批责任制和过错追究制，提高政府公信力。建立公务员信用承诺制度，形成高效、廉洁、诚信的行为规范。加强行业自律，建立企业信用评价、监督管理、责任追究等信用制度，促进企业守法经营。逐步建立个人信用联合征信制度，提高公民诚信意识。重视发挥新闻媒体和社会舆论的监督作用，加大失信惩罚力度，形成诚信为本、操守为重的良好社会风尚。

　　建立规范的市场经济秩序。加强土地、劳动力、技术等要素市场建设。完善对市场主体资格和市场行为等方面的政府规定。严厉打击制假售假、走私贩私、偷税骗税、逃汇骗汇和逃废债务等破坏市场经济秩序的违法犯罪活动。禁止地方封锁和行业垄断，依法规范市场准入和竞争行为。严肃查处做假帐和在建设工程招标中弄虚作假等腐败违法行为。加强地方金融资产管理。整顿规范文化市场、娱乐场所和互联网上网服务等营业场所。强化知识产权保护和管理。依法规范中介活动。

　　切实加强安全生产监督管理。全面贯彻《中华人民共和国安全生产法》，进一步落实安全生产责任制和重特大安全事故行政责任追究制度，推进注册安全主任制度。抓好安全生产专项整治，排除事故隐患，有效防止重特大事故发生，努力减少一般性事故的发生。在市区主干道设置摩托车专用道，减少交通事故。

　　高度重视维护社会稳定工作。落实社会治安综合治理目标管理责任制、领导责任制和一票否决制度。继续开展严打整治，严厉打击各种暴力犯罪、带黑社会性质的团伙犯罪、毒品犯罪以及抢劫、盗窃等多发性犯罪，集中整治治安混乱的重点地区和突出问题，完善打防控一体化的社会治安长效机制。严密防范、坚决打击敌对势力和“法轮功”邪教组织的破坏活动。坚决扫除“黄赌毒”等社会丑恶现象。加强司法行政工作。正确处理人民内部矛盾，重视人民群众来信来访，抓好矛盾纠纷排查调处工作，健全工作机制和责任制，及时发现和化解各种苗头性、倾向性问题，把问题解决在萌芽状态，解决在基层。依靠各方力量维护社会稳定。

　　十二、建设服务型政府，提高行政效率

　　加快政府职能转变。按照建立“行为规范、运转协调、公正透明、廉洁高效”的行政管理体制要求，进一步转变政府职能，转变工作方式，改进机关作风，继续抓好首问责任制和政务承诺制，完善投资服务中心工作，提高服务水平。推进电子政务，提高行政效率，降低行政成本。进一步深化行政审批制度改革，精简审批事项，规范审批程序，依法规范收费行为，推行相对集中行政处罚权工作，努力建设阳光政府、信用政府、服务型政府，树立良好的政府形象。

　　完善政务公开工作。增强政府工作的透明度、公开化，凡不涉及国家安全和保密以及法律法规规定的事项，政府机关作出的对社会公众具有普遍约束力的行政规定，事关全局的重大决策过程和出台的政策，以及群众普遍关心的事项等，都要通过适当的形式向社会公开，接受社会监督。

　　加强政风建设和廉政建设。落实“八个坚持、八个反对”，切实加强作风建设。精简会议和文件，把工作的着力点放在狠抓各项决策和工作部署的检查落实上。注重调查研究，经常深入基层了解情况，协调问题，帮助解决实际困难，多为基层和群众办好事实事。发扬艰苦奋斗优良传统，厉行节约，反对浪费，勤俭办事。深入开展廉政建设和反腐败斗争，落实廉政建设责任制，从决策和执行等环节加强对权力的监督，进一步从源头上预防和治理腐败。加强公务员队伍建设，提高公务员素质和服务水平。开展行风检查和评议工作，强化对政府机关及其工作人员的行政监察。在继续抓好政府承诺的实事落实的同时，今年要为市民办12件实事：（1）解决河朗、上洋、三山、平冈、海陵等5个镇部分群众饮水难问题；（2）建设市区部分特困户解困房；（3）抓好老区、山区60所小学改造；（4）抓好市区2所薄弱学校改造和市技工学校建设；（5）建好市劳动力中心市场；（6）建设市青少年活动中心；（7）设立市民营企业展示中心；（8）建设市疾病预防控制中心；（9）改造建设市区创业路北段、江台路、高凉路、石湾北路、洲尾路等道路；（10）抓好市区金鸡路、河堤南路、农科路的路灯建设；（11）改造市区第一市场；（12）启动金山森林公园首期建设工程。

　　各位代表！新的一年形势喜人，形势逼人，任务艰巨，催人奋进。让我们在以胡锦涛同志为总书记的党中央领导下，高举邓小平理论伟大旗帜，以“三个代表”重要思想为指导，全面贯彻党的十六大精神，进一步解放思想，实事求是，同心同德，奋发图强，扎实工作，与时俱进，为开创我市经济社会发展的新局面，推进全面建设小康社会而努力奋斗！
